# Pathophysiology and Advances in the Therapy of Cardiomyopathy in Patients with Diabetes Mellitus

**DOI:** 10.3390/ijms25095027

**Published:** 2024-05-05

**Authors:** Patryk Graczyk, Aleksandra Dach, Kamil Dyrka, Andrzej Pawlik

**Affiliations:** 1 Department of Physiology, Pomeranian Medical University, 70-111 Szczecin, Poland; patrykg1234@o2.pl (P.G.); dach.aleksandra@icloud.com (A.D.); 2 Department of Pediatric Endocrinology and Rheumatology, Institute of Pediatrics, Poznan University of Medical Sciences, 60-572 Poznan, Poland; kamil.dyrka@onet.eu

**Keywords:** diabetes mellitus, DM, diabetic cardiomyopathy, DCM, heart failure, metformin, SGLT2 inhibitors, GLP-1 analogues, thiazolidinediones, reactive oxygen species, inflammation, healthy lifestyle

## Abstract

Diabetes mellitus (DM) is known as the first non-communicable global epidemic. It is estimated that 537 million people have DM, but the condition has been properly diagnosed in less than half of these patients. Despite numerous preventive measures, the number of DM cases is steadily increasing. The state of chronic hyperglycaemia in the body leads to numerous complications, including diabetic cardiomyopathy (DCM). A number of pathophysiological mechanisms are behind the development and progression of cardiomyopathy, including increased oxidative stress, chronic inflammation, increased synthesis of advanced glycation products and overexpression of the biosynthetic pathway of certain compounds, such as hexosamine. There is extensive research on the treatment of DCM, and there are a number of therapies that can stop the development of this complication. Among the compounds used to treat DCM are antiglycaemic drugs, hypoglycaemic drugs and drugs used to treat myocardial failure. An important element in combating DCM that should be kept in mind is a healthy lifestyle—a well-balanced diet and physical activity. There is also a group of compounds—including coenzyme Q10, antioxidants and modulators of signalling pathways and inflammatory processes, among others—that are being researched continuously, and their introduction into routine therapies is likely to result in greater control and more effective treatment of DM in the future. This paper summarises the latest recommendations for lifestyle and pharmacological treatment of cardiomyopathy in patients with DM.

## 1. Introduction

Diabetes mellitus (DM) is one of the most common conditions in the world. It is characterised by a state of chronic hyperglycaemia in the body caused by a deficiency of insulin or reduced sensitivity of tissues to this hormone, leading to the impaired function of many organs, including the heart. DM is one of the causes of cardiomyopathy, which in this case is called diabetic cardiomyopathy (DCM) [1,2]. Numerous preclinical and clinical studies have been conducted to elucidate the pathophysiological mechanisms leading to cardiomyopathy. A state of chronic hyperglycaemia negatively affects cardiac metabolism through various mechanisms, including oxidative stress, chronic inflammation, the accumulation of advanced glycation end products (AGEs) and excessive activation of the hexosamine biosynthetic pathway. The aforementioned processes lead to cardiomyocyte hypertrophy, which results in the stiffening of the heart wall and impairment of its contractility and diastolic capacity. Antidiabetic drugs have different mechanisms of action, and an individualised approach to the patient is necessary, considering the comorbidities and potential side effects of the drugs. In this review, we describe the standard drug groups used in patients with DM and heart failure (HF), as well as alternative substances that may improve the treatment of DCM in the future. A drug has still not been developed that targets the lipotoxicity that damages cardiomyocytes in patients with DM. However, treatment with a sodium glucose cotransporter-2 (SGLT2) inhibitor is currently one of the most effective and recommended treatments for patients with DM and HF. In this review, we summarise the pathophysiology and treatment of cardiomyopathy in patients with DM. The present study was conducted by searching for the latest published studies (up to January 2024) available in PubMed, MEDLINE, Scopus and Google Scholar using the following key words: diabetes mellitus, diabetic cardiomyopathy, heart failure, inflammation, oxidative stress, cytotoxicity, hyperglycaemia, treatment and/or therapy of diabetic cardiomyopathy, alternative options for the treatment and/or therapy of diabetic cardiomyopathy and perspectives of diabetic cardiomyopathy treatment and/or therapy.

### 1.1. Historical Background

In 1972, Rubler et al. described cases of cardiomyopathy and death due to myocardial infarction in four patients diagnosed with DM but without other risk factors, such as hypertension, coronary atherosclerosis and valvular defects [1]. Thus, researchers hypothesised the existence of DCM, which they provided 5 years later [2]. The Framingham Study of 1979 showed an association between DM and a 2–3-fold increased risk of developing atherosclerosis, which can result in myocardial infarction [3]. This and subsequent studies have proved that patients with DM have a 1.5-fold increased risk of myocardial infarction compared with patients without DM [4,5]. These findings have inspired researchers to determine the mechanism by which pathological changes in the myocardium occur.

### 1.2. Epidemiology

In 2021, there were an estimated 537 million adults aged 20–79 years with DM throughout the world. In Poland, there were more than 2.6 million such patients. According to estimates, by 2045, the number of patients with DM will increase to more than 2.7 million in Poland and 783 million worldwide [6]. The World Health Organization (WHO) predicts that DM will become the seventh leading cause of death worldwide. It is believed that among patients with DM, death is most often due to cardiovascular complications, including HF [7].

### 1.3. Diabetes Mellitus and Heart Muscle

DM often runs concurrently with other disorders that increase the risk of myocardial infarction, including hypercholesterolemia, hyperlipidaemia, obesity, hypertension, thrombosis, ischaemia and coronary artery disease. The co-occurrence of the aforementioned conditions makes it impossible to determine which of them plays the greatest role in the pathomechanism of heart disease development [8]. The increased risk of myocardial infarction correlates with blood glucose levels, but in patients with DM, only chronic hyperglycaemia (blood glucose > 200 mg/dL) increases the risk of death from myocardial infarction [9]. A UK study showed that a 1% reduction in glycated haemoglobin (HbA1c) reduces the risk of myocardial infarction by 16% [10]. Therefore, it is extremely important to follow clinical recommendations to manage DM.

## 2. Diabetic Cardiomyopathy

**Figure 1:**
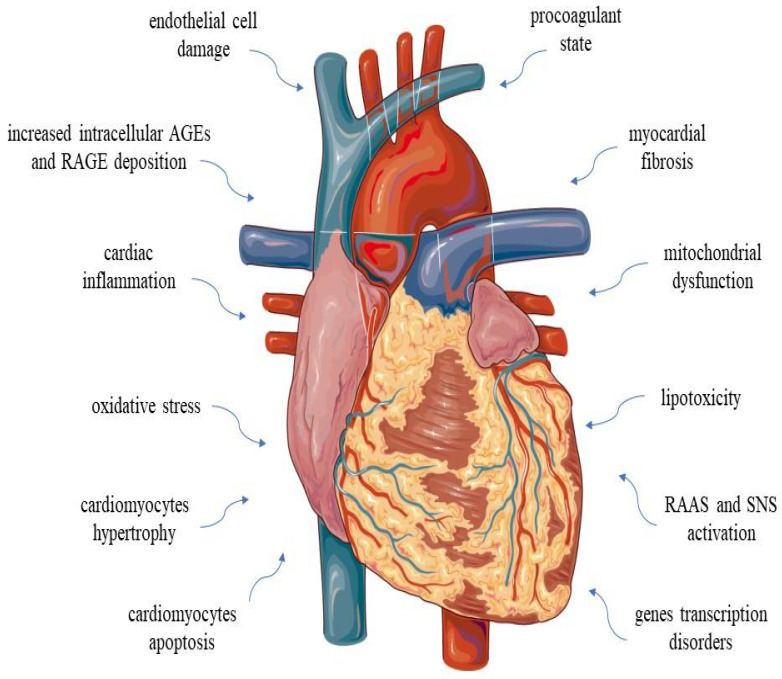
Multifactorial disorders in diabetic cardiomyopathy. Abbreviations: AGEs—advanced glycation end products; RAGE—receptor advanced glycation end product; RAAS—renin–angiotensin–aldosterone system; and SNS—sympathetic nervous system.

DCM is an organic heart disease resulting from abnormal myocardial structure and function in individuals with DM who do not have other conditions, such as coronary artery disease (CAD), hypertension, valvular heart disease (VHD) and congenital heart disease (CHD). DCM arises due to dysregulated glucose and lipid metabolism associated with DM, triggering the activation of various inflammatory pathways. Traditionally, DCM has been categorised into two stages. In the initial stage, DCM presents with metabolic disorders, autonomic dysfunction, impaired remodelling and concurrent diastolic dysfunction. Subsequently, cardiomyocyte fibrosis and apoptosis contribute to LV dilation, and symptomatic HF emerges with the development of systolic dysfunction. The diagnosis of DCM remains challenging due to the absence of a clear disease definition. During the early stage, patients with DCM are typically asymptomatic, while those in advanced stages may experience dyspnoea and/or peripheral oedema. The presence of congestive HF signs always requires thorough investigation [11,12]. DCM, a unique form of cardiovascular disease, can lead to the inability of the heart to circulate blood through the body effectively, a state known as HF [1,12,13]. As a result, the heart’s minute capacity is reduced relative to the metabolic demand of the body’s tissues or the proper minute capacity is maintained through increased filling pressures, resulting in clinical manifestations. We distinguish between newly diagnosed HF, transient HF and chronic HF. Another division defines the type of HF according to the left ventricular (LV) ejection fraction (LVEF): HF with a preserved LV ejection fraction (HFpEF), in which the LVEF is ≥50%; HF with a mildly reduced LV ejection fraction (HFmrEF), in which the LVEF is 41–49%; and HF with a reduced LV ejection fraction (HFrEF), in which the LVEF is ≤40%. HF can affect the LV, the right ventricle (RV) or both simultaneously [14]. HF is the most common cardiovascular cause of hospitalisation in people over 60 years of age [15]. The relationship between HF and DM is apparent: indeed, DM is an independent risk factor for HF. These disease entities are linked by a complication in the form of ischaemic heart disease and metabolic disorders such as glucose toxicity and lipotoxicity resulting from the insulin resistance of cardiomyocytes [16]. According to data from the last 25 years, the prognosis of patients with DM and HF is worse than that of patients without DM [17]. Patients with DM have a >3-fold increased risk of coronary ischaemic events and congestive HF. Most HF in people with DM results from CAD, and DCM is only said to exist if there is no CAD and other cardiac causes to explain the heart muscle disorder [18]. Figure 1 presents factors contributing to the development and progression of DCM.

## 3. Pathophysiology of Diabetic Cardiomyopathy—Metabolic Dyscontrol of Signalling Pathways

### 3.1. Myocardial Bioenergetics in the Diabetic Heart, Lipotoxicity and Increased Activity of the Renin–Angiotensin–Aldosterone System (RAAS)

**Figure 2:**
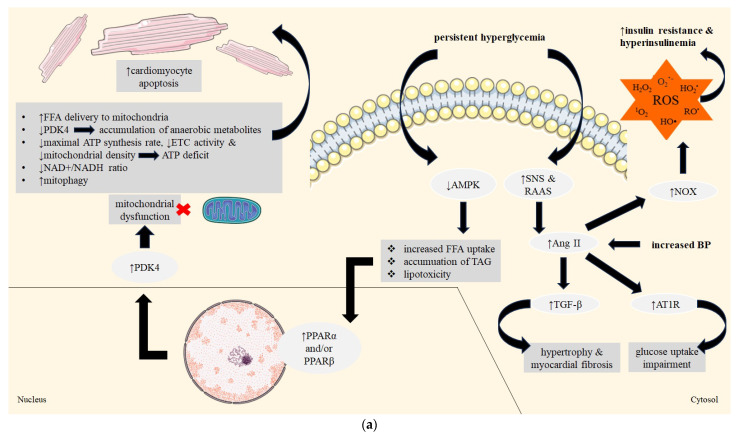

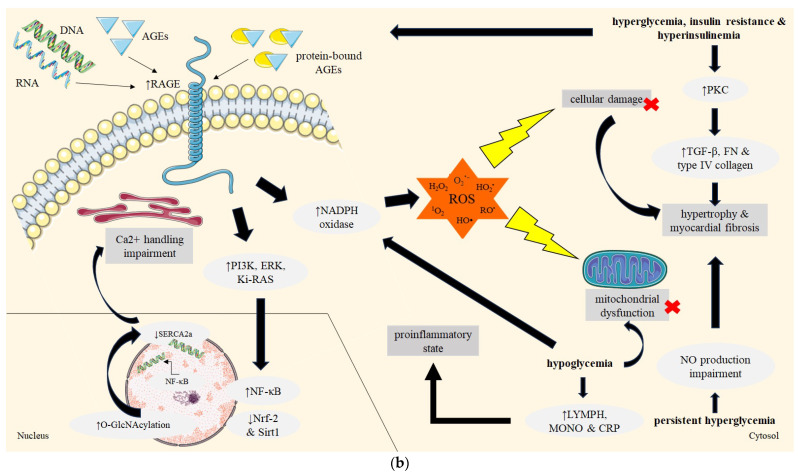
(**a**) Molecular proteins and signalling pathways in DCM—part 1. (**b**) Molecular proteins and signalling pathways in DCM—part 2. Abbreviations: TGF-β—transforming growth factor β; PI3K—phosphoinositide 3-kinases; ERK—extracellular signal-regulated kinase; Ki-RAS—Kirsten RAS; SERCA2a—sarcoendoplasmic reticulum Ca^2+^-ATPase 2a; NF-κB—nuclear factor kappa B; Nrf-2—nuclear factor erythroid 2-related factor 2; Sirt 1—sirtuin 1; RNA—ribonucleic acid; DNA—deoxyribonucleic acid; AGEs—advanced glycation end products; RAGE—receptor advanced glycation end product; ROS—reactive oxygen species; O_2_^•−^—superoxide ion; HO_2_^•^—hydroperoxyl radical; HO^•^—hydroxyl radical; RO^•^—alkoxy radical; ^1^O_2_—singlet oxygen; H_2_O_2_—hydrogen peroxide; NADPH—nicotinamide adenine dinucleotide phosphate; LYMPH—lymphocyte; MONO—monocyte; CRP—C-reactive protein; PKC—protein kinase C; FN—fibronectin; and NO—nitric oxide.

For energy acquisition, the myocardium uses mainly FFAs; very little glucose is used as a metabolic substrate. In patients with DM, these proportions are disrupted due to tissue insulin resistance as well as elevated blood FFA concentrations resulting from increased lipolysis in adipose tissue. The initiation of glucose uptake and glycolysis, crucial for improving cardiac function in diabetic patients, is mediated by the activation of AMP-activated protein kinase (AMPK). Nevertheless, in DM, AMPK activation becomes subdued, leading to increased FFA uptake, accumulation of triacylglycerol (TAG) and diminished glucose utilisation, all characteristic features of DCM. Cardiomyocytes adapt to the conditions in the body and increase the activity of the mitochondrial proteins responsible for FFA metabolism [19]. This adaptation requires increased transcription of genes that encode enzymes involved in this process, which occurs as a result of an increase in the activity of peroxisome proliferator-activated receptors type α and/or β (PPARα and/or PPARβ) [20]. An increase in PPARα activity results in greater pyruvate dehydrogenase kinase type 4 (PDK4) activity, which translates to a decrease in metabolic glucose consumption and an increase in FFA delivery to the mitochondria. FFAs decrease the activity of PDK4, which is responsible for aerobic glucose metabolism, resulting in the accumulation of anaerobic glucose metabolites that are a potential cause of cardiomyocyte apoptosis [21]. The metabolism of FFAs is less efficient (2.33 adenosine triphosphate [ATP] molecules per oxygen atom) than the aerobic metabolism of glucose (2.58 ATP molecules per oxygen atom), which puts even more strain on the myocardium. Moreover, in patients with DM, the myocardium is often forced to do even more work due to the comorbidities in these patients (i.e., overweight or obesity). When the transport of FFAs into the myocardium is greater than the ability to metabolise them by oxidation, they accumulate in cardiomyocytes as fat deposits and lipid droplets, indicating the lipotoxic effects of FFAs [22]. Their excess is metabolised via the anaerobic pathway to, among other things, ceramides, the accumulation of which can lead to premature apoptosis of cardiomyocytes [23]. An imbalance in the uptake and oxidation of FFA results in mitochondrial dysfunction. This process involves a reduction in several parameters within the myocardium, including the maximal ATP synthesis rate, expression and activity of the electron transport chain (ETC) and mitochondrial density. Conversely, there is an elevation in reactive oxygen species (ROS) production in endothelial cells and adipocytes. Mitophagy, a form of autophagy, serves a protective function in DCM by clearing abnormal mitochondria. Nevertheless, excessive mitophagy may intensify myocardial damage in individuals with DCM [24,25]. Protein hyperacetylation due to a decreased NAD+/NADH ratio intensifies diabetic heart metabolism insufficiency. Clinical studies have shown that individuals with type 2 DM (DM2) who engage in exercise exhibit an ATP deficit and inadequate oxygenation of the heart [26,27]. DM is associated with changes in the glomerular filtration rate, mass and kidney function, which leads to increased blood pressure. DM significantly alters the body’s endogenous water and electrolyte balance. Chronic hyperglycaemia promotes excessive activation of the sympathetic nervous system (SNS) and the renin–angiotensin–aldosterone system (RAAS). Studies indicate that increased blood pressure is associated with increased serum levels of angiotensin-converting enzyme (ACE) in patients with DM. As the disease progresses, increased angiotensin II (Ang II) activity causes hypertrophy of the mesangial cells and renal tubular epithelial cells. Ang II also promotes the production of the pro-sclerotic cytokine TGF-β, which is one of the causes of glomerular sclerosis [28]. Ang II interferes with glucose uptake and induces alterations in blood flow. Recent studies suggest that a decrease in the phosphorylation of glycogen synthase kinase 3 beta (GSK-3β) impairs glucose uptake. The activation of the angiotensin 1 receptor (AT1R) restricts the microvascular blood volume and hinders glucose extraction. An elevated concentration of Ang II is linked also to the activation of NOX, resulting in ROS generation and a rise in insulin resistance [29]. Figure 2a summarises the mechanisms of lipotoxicity, mitochondrial dysfunction and increased activation of the RAAS and SNS in DCM.

### 3.2. Glucotoxicity in Diabetic Cardiomyopathy

Uncontrolled hyperglycaemia damages tissues through several mechanisms. An important role is played by increased production of intracellular AGEs and increased expression of receptor advanced glycation end products (RAGEs), as well as activation of the polyol or hexosamine pathway. In endothelial cells, the AGE–RAGE complex modulates the expression of several genes, including those for thrombomodulin, tissue factor and vascular cell adhesion molecule-1 (VCAM-1). As a result, there are procoagulative changes on the surface of endothelial cells and increased adhesion of proinflammatory factors [30,31]. AGEs directly damage cells and increase ROS production. Extracellular AGEs via specific receptors (RAGEs) activate NADPH oxidase (NOX). There are seven isoforms of NOX expressed in mammals, and two of them—NOX2 and NOX4—exist in the heart. Their primary function is to generate ROS, potentiating the inflammatory process and oxidative stress [32].

High glucose levels affect the body’s inflammatory balance, as it inhibits antioxidant pathways mediated by nuclear factor erythroid 2-related factor 2 (Nrf2) and sirtuin 1 (Sirt1). In turn, it stimulates the proinflammatory response by activating nuclear factor κB (NF-κB) [33]. Moreover, hyperglycaemia in patients with DM impairs calcium handling in cardiomyocytes by increasing nuclear O-GlcNAcylation, which results in decreased expression of messenger RNA (mRNA) encoding a sarcoendoplasmic reticulum protein called sarcoendoplasmic reticulum Ca^2+^-ATPase 2a (SERCA2a) and decreases SERCA2a promoter activity. The study showed that in isolated, prefunded animal hearts, increased O-GlcNAcylation inhibits phenylephrine-induced inotropy due to impaired capacitive Ca^2+^ influx through membrane channels activated in response to the depletion of sarcoendoplasmic reticulum Ca^2+^ stores [34].

On the other hand, hypoglycaemia is also not beneficial for heart function. Hypoglycaemia is defined as a blood glucose concentration < 70 mg/dL [35]. This condition activates the sympathoadrenal system and causes the release of the catecholamines. As a result, the heart rate, systolic blood pressure, cardiac output and ejection fraction increase and the diastolic blood pressure decreases [36]. In patients with DM2, the hypoglycaemia-induced heart rate variability response is impaired, so they are at higher risk of developing arrhythmia [37]. Hypoglycaemia can also affect cardiac repolarisation and electrophysiology, which can be seen in ECG scans as ST segment depression, changes in the T wave and QT interval prolongation [38]. It is the QT interval prolongation (in response to hypokalaemia induced by the catecholamines) that increases the risk of ventricular arrhythmia and may lead to sudden death [39]. Hypoglycaemia may also result in increased ROS generation and decreased activity of mitochondrial antioxidant enzymes, causing oxidative stress in cells. Moreover, the numbers of lymphocytes and monocytes, C-reactive protein (CRP) and other inflammatory proteins rise in response to a blood glucose concentration < 70 mg/dL resulting in the proinflammatory state [40].

### 3.3. Generation of Reactive Oxygen Species (ROS), Fibrosis and Chronic Inflammation

Oxidative stress plays a key role in the pathogenesis of microvascular complications in DM. ROS and inflammatory factors promote and exacerbate hypertrophy and myocardial fibrosis [8,41,42]. Excess glucose stimulates mitochondrial superoxide production as it is reduced to sorbitol by aldose reductase, which reversibly binds NADPH, in the polyol pathway. That impairs glutathione reduction and resultantly contributes to oxidative stress [42]. In addition, there is excessive activation of several protein kinase C (PKC) isoforms that mediate tissue damage. PKC can enhance microvascular matrix protein accumulation by inducing the expression of transforming growth factor β1 (TGF-β1), fibronectin and type IV collagen in mesangial cells [30]. Impaired NO production also results in the activation of collagen cross-linking enzymes and promotes cardiac fibrosis [8]. DM causes chronic inflammation, which is mediated by increased inflammasome activity [16]. The nucleotide-binding oligomerisation domain-like receptor protein 3 (*NLRP3*) inflammasome is associated with metabolic disturbances and cell death that contribute to the development of DCM [33]. *NLRP3* is activated by hyperglycaemia and high concentrations of FFAs. *NLRP3* activation induces the production of interleukin 1β (IL-1β) and IL-18 and induces local tissue inflammation [16]. NF-κB and thioredoxin interacting/inhibiting protein (TXNIP) mediate ROS-induced activation of caspase-1 and IL-1β. These compounds are effectors of the *NLRP3* inflammasome. The phenomenon of pyroptosis, a type of programmed lytic cell death mediated by caspase-1 of the *NLRP3* inflammasome, has been found in the myocardium of diabetic rats. NF-κB induces the production of TNF-α (tumour necrosis factor alpha), which is indirectly cytotoxic. Silencing the *NLRP3* gene may have a cardioprotective effect and participate in the treatment of DCM [43]. Figure 2b summarises the mechanisms of glucotoxicity, ROS generation, increased fibrosis and chronic inflammation in DCM.

## 4. Treatment of Diabetic Cardiomyopathy

### 4.1. Non-Pharmacological Treatment

In the early stages of DCM, impaired cardiac contractile capacity and cardiomyocyte hypertrophy are reversible. In contrast, as the disease progresses and with advanced structural changes in cardiomyocytes, no current treatment regimen has a fully satisfactory therapeutic effect. Treatment is multidirectional and includes non-pharmacological management and pharmacotherapy. Normalisation of body weight; lifestyle modification; and control of the parameters of carbohydrate metabolism (HbA1c), the lipid profile and blood pressure are crucial. The American and European Heart Association guidelines now recommend a so-called ‘heart healthy diet’. Among these recommendations is an emphasis on properly adjusting caloric intake and eating unprocessed poultry, fresh fish, nuts, legumes and a variety of vegetables. Indeed, the substitution of red and processed meat has been associated with a lower risk of total mortality and cardiovascular deaths [16,44,45]. Two to three servings of fish per week have been shown to be associated with lower mortality from any cause or lower risk of HF [46]. In addition, it is recommended to limit consumption of sweetened beverages and highly processed foods containing high amounts of salt and to reduce or stop alcohol consumption [44,45]. The most common diets considered in patients with DM are the Dietary Approaches to Stop Hypertension (DASH) diet, the Mediterranean diet and the vegetarian/vegan diet. However, an individualised approach to the patient that includes collaboration between the physician and dietitian to select the most optimal dietary plan is essential [47].

Physical activity is an important non-pharmacological strategy for reducing the complications of DM and CVRFs. Physical activity causes adaptation of the heart as well as the vessels. It protects the heart from ischaemia-induced damage, modulates myocardial metabolism and prevents excessive cardiomyocyte hypertrophy. Exercise effectively prevents apoptosis, cardiac fibrosis and haemodynamic disorders of the microcirculation caused by high glycaemic levels. In addition, exercise effectively lowers blood glucose levels, increases tissue sensitivity to insulin, reduces oxidative stress, benefits cardiac calcium metabolism and increases the number of mitochondria, resulting in increased myocardial contractile force [48,49]. Table 1 summarises the dietary and physical activity recommendations for patients with DCM.

### 4.2. Pharmacological Treatment Established Therapies of Diabetic Cardiomyopathy Anti-Hypertensive Drugs

#### 4.2.1. Angiotensin-Converting Enzyme Inhibitors and Angiotensin Receptor Blockers

In patients with DM2, adipocytes secrete many hormones, including Ang II. Excess Ang II has a stimulatory effect on the inflammatory process that is already occurring in the body and increases oxidative stress in tissues [55]. Moreover, this hormone contributes to the progression of DM by increasing insulin resistance [56]. An extremely important action of ACE inhibitors (ACEIs) and angiotensin receptor blockers (ARBs) is the inhibition of collagen synthesis and stimulation of matrix metalloproteinase (MMP) activity. Thus, these drugs slow down myocardial remodelling and reduce the risk of developing myocardial fibrosis and LV stiffness. Moreover, they inhibit cardiomyocyte hypertrophy and the development of LV hypertrophy (LVH) [55,56,57,58]. Recommendations unequivocally point to ACEIs as first-line therapy in patients with HF. Numerous randomised controlled trials (RCTs) have shown that an ACEI reduces overall mortality and cardiovascular mortality in patients with DM and overt HF, as well as in patients with asymptomatic LV dysfunction [57]. By blocking ACE, the enzyme that converts Ang I to Ang II, an alternative pathway for regulation of the renin–angiotensin axis is unblocked. During the use of ACEIs, another enzyme—ACE2—is activated; it converts Ang I into other forms of angiotensin, namely, Ang (1–7). Reducing the amount of Ang II and increasing Ang (1–7) contributes to improving the diastolic capacity of the heart, because as blood vessels dilate, the load on the heart decreases. Therefore, drugs that stimulate alternative regulation of the renin–angiotensin axis—including type 1 Ang II receptor antagonists and neprilysin inhibitors (ARNis)—may be the future of DCM therapy [29,56,57].

A representative ARNi is a 1:1 ratio of sacubitril (AHU 377, a neprilysin inhibitor) and valsartan, an ARB; the preparation is also known as LCZ696 [29,56]. This ARNi inhibits excessive activation of the RAAS system and thus has a protective effect on the cardiovascular system by, among other things, increasing natriuretic peptide levels [58]. Animal studies have been conducted to evaluate the effect of ARNis on the mechanisms that cause the development of DCM. The study showed that LCZ96 inhibits cell apoptosis by, among other things, decreasing the expression of caspase-3 and the Bax/Bcl-2 ratio. In addition, LCZ96 reduces ROS and oxidative stress induced by hyperglycaemia. In mice with DM, LCZ96 counteracts the decrease in reduced glutathione (GSH) levels and the cellular GSH-to-oxidised glutathione (GSSG) ratio. LCZ96 exerts an anti-inflammatory effect by significantly reducing proinflammatory cytokines, such as IL-1β, IL-6 and TNF-α [59].

#### 4.2.2. Beta-Blockers

Beta-blockers prevent and reverse structural and functional changes in HF and protect against the toxic effects of catecholamines. In addition, they have antioxidant and anti-endothelin effects, which translate into the effective prevention of LV dysfunction [60]. Long-term beta-blocker therapy can improve LVEF by increasing the stroke volume, reducing pulmonary capillary and right atrial pressures and decreasing systemic vascular resistance [61]. A representative beta-blocker is carvedilol, which has an antiadrenergic effect and also reduces sympathomimetic effects in patients with DCM [60]. Another drug in this group is nebivolol, which is a highly selective β1-adrenoceptor blocker that also increases the bioavailability of NO in the L-arginine–NO pathway, leading to vasodilation and decreased vascular resistance [62]. Beta-blockers reduce the risk of HF hospitalisation and mortality in patients with HFrEF, although in patients with HFpEF, the use of beta-blockers is associated with an increased risk of complex cardiovascular events, a higher risk of death from any cause and HF-related hospitalisation [63,64]. Additional studies are needed to reveal the indications for the use of beta-blockers in patients with DCM.

#### 4.2.3. Mineralocorticoid Receptor Antagonists

All mineralocorticoid receptor antagonists (MRAs) have highly similar mechanisms of action regarding DCM therapy: they improve cardiac function and can be an important part of the treatment of this condition. Aldosterone antagonists include spironolactone, eplerenone, canrenone and finerenone. Researchers have shown that blocking the aldosterone receptor has a beneficial effect on cardiac metabolism. Eplerenone delays cardiomyocyte apoptosis and cardiomyocyte steatosis. It also reduces fibrosis and hypertrophy of cardiac muscle, improves the diastolic capacity and improves cardiomyocyte energy processes by reducing cellular insulin resistance and ROS production, which reduces inflammation [65]. Similar effects have been observed with spironolactone. In an animal study, the researchers found that spironolactone reduces fibrosis and hypertrophy of cardiomyocytes and reduces oxidative stress by decreasing ROS production, resulting in improved mitochondrial function and increased efficiency of energy processes [66]. Finerenone is a third-generation MRA that also has proven beneficial effects on the cardiovascular system in patients with DM2. In contrast, its effect on DCM has yet to be clearly defined. An animal study identified possible mechanisms for the drug’s beneficial effects in DCM. Finerenone attenuates fibrosis and apoptosis of cardiomyocytes without affecting blood glucose levels. As a result, it prevents cardiac hypertrophy and has a significant effect on the diastolic capacity of the heart. It also significantly improves the metabolism of FFAs in the heart, reducing their accumulation in cardiomyocytes and decreasing their degree of steatosis [67].

#### 4.2.4. Sodium Glucose Cotransporter-2 Inhibitors

SGLT2 inhibitors are one of the main drug groups that are used in patients with established HF and/or chronic kidney disease. RCTs have shown that these drugs reduce the risk of cardiovascular death and hospitalisations for diabetic and non-diabetic HF, irrespective of ejection fraction or care setting [68,69]. SGLT2 facilitates the movement of glucose and sodium across the cell membrane in proximal renal tubules [70]. Inhibition of SGLT2 results in insulin-independent improvement in glycaemic control due to glucose excretion. SGLT2 inhibitors also reduce the lipid accumulation in cardiomyocytes and lower blood insulin levels and tissue insulin resistance [16,68,69,70,71,72]. They are also said to act directly on pancreatic α-cells increasing plasma glucagon levels. As a result, the ratio of insulin to glucagon decreases, leading to lipolysis and excessive production of FFAs, which are metabolised in the liver to ketones: acetoacetate, β-hydroxybutyrate (β-OHB) and acetone. These metabolic changes predispose patients to ketoacidosis while preserving normoglycaemia [73]. It has been observed that starting an SGLT2 inhibitor treatment resulted in approximate doubling in serum fasting ketones levels [74]. The study run by Nielsen et al. presented that β-OHB has beneficial dose-dependent haemodynamic effects in patients with HFrEF, such as increased stroke volume, cardiac output and LVEF [75]. What is more, β-OHB inhibits the *NLRP3* inflammasome and attenuates inflammatory responses [76]. β-OHB is also an endogenous inhibitor of histone deacetylases, the inhibition of which correlates with increased transcription of the genes encoding oxidative stress resistance factors [77]. In that way, ketone bodies may contribute to the reduction in oxidative stress. Due to their natriuretic effects, there is also a reduction in blood pressure and body weight [16,68,69,70,71,72]. Optimisation of the hydration status does not increase the heart rate and may even improve myocardial contractility. The likely mechanism is a reduction in arterial stiffness and a decrease in vascular resistance of the feeding arterioles in the kidneys [71]. In addition, SGLT2 inhibitors may indirectly activate signalling pathways such as AMPK or SIRT1 and consequently reduce oxidative stress and cellular inflammation [72]. A preclinical study in mice suggested that cardioprotection results from the reduction in oxidative stress, fibrosis and DCM progression through the inhibition of inflammation-modulated cell lysis involving the NLPR3 inflammasome [78]. Empagliflozin reduces the oxidative stress by depleting the expression of NOX4, the NADPH oxidase isoform in cardiomyocytes associated with cardiomyopathy. Data suggest that empagliflozin also inhibits myocardial fibrosis by inhibiting the TGF-β/Smad pathway, which results in reducing the expression of TGF-β1 and collagen I and collagen III proteins [79]. Similarly, a study on mouse models showed that dapagliflozin possibly attenuates fibrosis, reduces inflammation and improves systolic function of diabetic hearts. In vitro experiments suggest that dapagliflozin may reduce calcium entry and attenuate oxidative stress associated with high glucose and angiotensin, apparently regardless of glycaemic control [79].

Potential targets in the therapy of diabetic cardiomyopathy.

Antidiabetic and anti-hyperlipidemic drugs.

#### 4.2.5. Incretin-Based Therapies

(a) Glucagon-like peptide-1 agonists

Glucagon-like peptide-1 (GLP-1) is an incretin hormone secreted by the L cells of the small intestine. There are GLP-1 receptors in the central nervous system and the gastrointestinal tract, more specifically in the intestine and the endocrine part of the pancreas [80]. GLP-1 secretion is induced by consuming a meal, especially one rich in carbohydrates. GLP-1 stimulates the release of insulin while inhibiting the release of glucagon and thus prevents the rise in postprandial blood glucose [81]. Naturally occurring GLP-1 has a very short half-life, so synthetic analogues with longer half-lives are used for treatment. They are widely used especially in patients with obesity, in whom they improve glycaemic control and promote weight loss [48]. Preclinical studies on animals have shown that GLP-1 agonists may reduce the risk of adverse cardiovascular events in patients with DM2. Exenatide is a synthetic equivalent of exendin-4, a compound that arrests myocardial remodelling after myocardial infarction by inhibiting the activation of β-catenin and stimulating the action of glycogen synthase kinase-3, β-arrestin-2 and protein phosphatase 2A [82]. By increasing antioxidant concentrations, exenatide-4 prevents episodes of myocardial ischaemia and attenuates myocardial fibrosis by inhibiting collagen deposition in rats’ hearts [82,83]. Exenatide-4 improves the sensitivity of cardiomyocytes to insulin. Another drug in this group, liraglutide, increases nitric oxide (NO) bioavailability and inhibits eNOS S-glutathionylation, thereby reducing oxidative stress in the mice’s vascular endothelium [84]. However, more clinical research still needs to be conducted confirming these results.

There are several mechanisms by which GLP-1 agonists may have therapeutic potential for inhibiting DCM progression. By stimulating the activity of catalase and manganese superoxide dismutase (MnSOD), the release of proinflammatory cytokines, caspase-1, TXNIP and ROS is inhibited, resulting in reduced inflammation in cardiomyocytes. In addition, by blocking RAGEs, GLP-1 agonists inhibit hyperglycaemia-induced cell apoptosis. Meanwhile, in the cell culture of cardiomyoblasts, GLP-1 agonists have an inhibitory effect on the p53 protein and thus delay apoptosis. GLP-1 agonists can block C/EBP homologous protein (CHOP)-induced endoplasmic reticulum (ER) stress by delaying the unfolded protein response (UPR) in the ER. In addition, these drugs may reduce cardiomyocyte damage and glucose toxicity by promoting autophagocytosis, which is associated with the inhibition of the phosphorylation of the mechanistic target of the rapamycin (mTOR) signalling pathway and the induction of AMPK phosphorylation [85].

However, a meta-analysis of RTCs has shown that GLP-1 agonists do not significantly reduce HF hospitalisation. Although, diastolic function measured using cardiac ultrasound was significantly improved [86]. It was suggested that GLP-1 agonists may even increase the risk of HF hospitalisation in patients with HFrEF (LVEF <40%) [87]. More dedicated RCTs evaluating the effects of GLP-1 RA in patients with diabetes and HF are needed [86,87].

GLP-1 agonists are usually well tolerated by patients. Among this group of drugs, liraglutide and exenatide are the ones more likely to cause side effects. Pancreatitis is the most commonly reported gastrointestinal side effect, followed by nausea and vomiting [88]. GLP-1 agonists are also known to increase heart rate. However, it should not increase the cardiovascular risk of individuals with DM2 and those at high risk of or with CVD [89].

(b) Dipeptidyl peptidase-4 inhibitors

Dipeptidyl peptidase-4 (DPP-4) is the enzyme that breaks down endogenous GLP-1. Inhibitors of this enzyme increase the half-life, and thus the action, of GLP-1. This group of drugs includes sitagliptin, vildagliptin, linagliptin, saxagliptin, alogliptin and evogliptin, among others. These drugs are used as DM2 monotherapy or, more often, in combination with other DM medications. By inhibiting gastric emptying and thus promoting weight loss, they are particularly desirable in patients with obesity [48]. They effectively control glycaemia without the risk of hypoglycaemic episodes. These drugs also have the advantage of being well tolerated: the side effects, such as diarrhoea and upper respiratory tract infections, are usually mild [90]. Numerous studies have been conducted to evaluate the effects of this group of drugs on the cardiovascular system. Saxagliptin, sitagliptin, linagliptin and alogliptin have been shown to be safe for therapy but do not reduce the risk of cardiovascular diseases (CVDs) and are therefore not recommended in patients with stage B or C HF [91]. In addition, saxagliptin may increase the risk of hospitalisation for HF [92]. Sitagliptin can enhance glucose uptake by cardiomyocytes in patients with non-ischaemic cardiomyopathy, a phenomenon that is desirable in stressful situations for the heart (under normal conditions, FFAs are the primary energy substrate). The inability of myocardial cells to utilise glucose under stressful conditions is one of the reasons for the development of DCM [93]. The results of a study published in 2023, in which the study group was obese mice with DM, showed that evogliptin can have a positive effect on cardiac function and structure and prevent DCM. In addition, the group of patients receiving the drug showed improved systolic and diastolic function, reduced cardiomyocyte hypertrophy and fibrosis and decreased lipid accumulation and thus lipotoxicity in myocardial cells [94]. In summary, the current recommendations indicate that DPP-4 inhibitors should not be the treatment of choice in patients with known CVD or multiple risk factors, except when newer antidiabetic drugs (GLP-1 agonists and SGLT2 inhibitors) are not tolerated, contraindicated or unavailable to the patient [95].

#### 4.2.6. Sulphonylurea Derivatives

Sulphonylurea derivatives have long been one of the main pillars of DM2 therapy due to their good antihyperglycaemic effect, low treatment cost, good tolerance by patients and mild side effects, apart from hypoglycaemic episodes [96]. Recently, sulphonylureas have been gradually displaced by newer drugs, including GLP-1 agonists and SGLT2 inhibitors, due to their low risk of inducing hypoglycaemia and their benefits in patients with obesity or renal and cardiac conditions [97]. Also contributing to the changes in the therapy regimen are reports of the cardiovascular effects of sulphonylureas, although these effects are unclear due to the proprietary nature of the studies. The cardiovascular effects may occur due to the different binding affinities of sulphonylurea derivatives for SUR subtypes. The ATP-sensitive potassium channel (K_ATP_ channel) is an octamer composed of four Kir6 subunits and four SUR subunits. There are two SUR isoforms, SUR1 and SUR2, which have two variants—SUR2A and SUR2B [98]. SUR1 is most abundantly expressed in the brain and pancreas, whereas SUR2A is highly expressed in cardiac tissue and SUR2B is expressed in vascular smooth muscle cells [99]. The sarcolemmal K_ATP_ channel in the heart may play a crucial role in the process of ischaemic preconditioning, the phenomenon in which a previous, short ischaemic event results in subsequent protection against a later, more severe ischaemic event. K_ATP_ channels are vital in the myocardial response to stress [100]. Sulfonylureas derivatives induce β-cell K_ATP_ channel closure on binding to SUR1, thus causing insulin secretion [101]. Different molecules among this type of drug have different binding affinities for SUR subtypes. Given that, it is possible that sulfonylureas derivatives cause adverse cardiac outcomes due to their ability to close the K_ATP_ channel in cardiac tissue, as this prevents the protective effects of ischaemic preconditioning [102]. One study found no significant difference in the incidence of HF among patients with DM2 treated with sulphonylureas versus insulin [103]. More than 20 years later, however, researchers observed an increased risk of death and hospitalisation for HF in patients with DM2 using sulphonylureas regardless of the ejection fraction [104]. On the other hand, the CAROLINA trial showed that there is a similar risk of cardiovascular events among adults with DM2 who use glimepiride or linagliptin, which was an interesting outcome [105]. The reason for this may be due to the fact that the study was designed to include patients at lower cardiovascular risk so that the results could be easily applied to patients in clinical practice. It has to be pointed out that the results of this trial should not be extrapolated to other sulphonylurea derivatives [106]. In view of the above, sulphonylureas are not recommended for patients with stage B or C HF [107].

#### 4.2.7. Insulin

Insulin administration is the mainstay of treatment for patients with type 1 diabetes mellitus (DM1) and patients with advanced DM2 when other medications fail to compensate for hyperglycaemia. Insulin treatment covers approximately 30% of patients diagnosed with heart failure and diabetes. Insulin supply to maintain glycaemia within the reference range can mitigate the progression of DCM [48]. There is a reduced risk of macro- and microangiopathic complications during intensive insulin treatment in patients with DM1 [108]. In contrast, Shen et al. showed that insulin use in patients with DM2 and HFpEF was associated with higher LV end-diastolic pressure and more severe LV diastolic dysfunction than in patients not treated with insulin [109]. Insulin leads to sodium and water retention, weight gain and hypoglycaemia and ultimately activation of the sympathetic nervous system [110]. Staszewsky et al. paid particular attention to the dark side of insulin treatment in patients with DCM. Insulin treatment of DM in patients with HF increases the risk of hospitalisation and death due to HF [111]. The daily dose of insulin should be properly adjusted to minimise that risk and alleviate the adverse effects. It has been proven that adding an SGLT2 inhibitor as well as a GLP-1 agonist to insulin therapy significantly reduces the risk of death, renal failure, atrial fibrillation, peripheral vascular diseases and adverse cardiovascular events in patients with DCM. As mentioned before, the use of SGLT2 inhibitors has a positive impact on reducing the risk of heart attack and hospitalisation due to HF. In turn, GLP-1 agonists are associated with significantly better stroke prevention [111]. In addition, both groups of drugs allow for a reduction in insulin dosage due to their hypoglycaemic effects. Adding insulin, if necessary, to the patient’s treatment regimen requires an individual approach and the consideration of many factors when adjusting the proper dosage.

#### 4.2.8. Thiazolidinediones

The thiazolidinediones (TZDs) currently used for patients with DM2 are pioglitazone and rosiglitazone. Treatment with TZDs is associated with a reduction in both fasting and postprandial blood glucose. Pioglitazone and rosiglitazone significantly inhibit the progression of the prediabetic state to DM [112]. One study showed that TZDs prevent mitochondrial degeneration and inhibit pancreatic beta-cell apoptosis and excessive lipid accumulation in obese prediabetic rats, resulting in improved cellular sensitivity to insulin [113]. TZDs have antioxidant and anti-inflammatory effects: they scavenge ROS, reduce plasma and cardiomyocyte FFA levels and inhibit activation of the proinflammatory factor NF-κB. Moreover, TZDs inhibit vascular smooth muscle proliferation and hypertrophy of the coronary intima [114].

As PPARγ agonists, they activate sodium transporters in the renal proximal tubule and endothelial sodium channels in the collecting duct, resulting in sodium and water retention. This creates a risk of oedema and increased volume loading on the heart. Many RCTs showed that TZDs are associated with an increased risk of developing congestive HF. This is more likely due to the water retention rather than a direct effect on the myocardium [115]. TZDs are not recommended for patients with a cardiac history because they increase the risk of myocardial infarction [22]. In conclusion, the data suggest that TZDs should be avoided in patients with NYHA class III and IV and used cautiously in patients with NYHA Stage I and II [116].

#### 4.2.9. Metformin

Metformin improves peripheral insulin sensitivity and reduces hepatic glucose secretion, which greatly promotes the control of hyperglycaemia [48]. One mechanism of action of metformin is the stimulation of adenosine monophosphate-activated protein kinase (AMPK). This enzyme regulates energy metabolism in various tissues of the body, including the heart, liver and muscle. Animal studies have been conducted confirming the beneficial effects of metformin on their cardiac muscle. In mice with HF caused by ischaemia, the drug improves LV function and prolongs survival [117]. When administered to dogs, metformin significantly improves myocardial performance [118]. The drug has also been shown to increase cardiomyocyte autophagy, which protects against the development of DCM in animal models [119]. There is a lack of prospective RCTs evaluating whether metformin can be a first-line drug in patients with DCM. Of note, several months of metformin therapy increases the risk of vitamin B12 and folic acid deficiency and may contribute to the progression of diabetic neuropathy, so monitoring for deficiency and supplementing these vitamins in patients is extremely important [120].

Anti-hyperlipidemic drugs.

#### 4.2.10. Statins

Statins are believed to have a protective role in treating DCM due to their anti-inflammatory effects. In addition to their lipid-lowering effects, statins exert inhibitory and/or stimulatory effects on the *NLRP3* inflammasome and TLRs. It can suppress TLR4/MyD88/NF-ĸB signalling and shift to an anti-inflammatory response. It inhibits the NF-ĸB pathway by decreasing the expression of TLR2 and TLR4. Atorvastatin improves LV function by reducing myocardial inflammation and fibrosis and increasing NO availability. Furthermore, Rosuvastatin also exhibited protective properties by reducing *NLRP3* and IL-1β inflammasome activation through the suppression of the MAPK pathways. Simvastatin reduces myocardial dysfunction by attenuating hyperglycaemia-induced cardiac oxidative stress, inflammation and apoptosis [121,122].

#### 4.2.11. Fibrates

Fibrates are anti-hyperlipidemic drugs that target PPAR-α as their agonists. As a result, they stabilise serum TAG levels and inhibit excess lipids metabolism. Their mechanism of upregulating FFA metabolism consists of five major points. Fibrates induce lipolysis of lipoproteins and hepatic FFA uptake. They reduce TAG production in the liver. Moreover, they increase LDL catabolism and HDL production. They also stimulate reverse cholesterol transport [123]. Fenofibrate has been shown to mildly reduce fibrosis in a diabetic rat’s heart as it decreases lipid accumulation in cardiomyocytes [124]. These findings suggest that treatment with fibrates may be beneficial for patients with DCM.

#### 4.2.12. Modern and Possibly Effective Treatments

Research into modern and effective treatments for DCM is ongoing. DM is a progressive disease; thus, patients usually require the addition of a new drug. Scientists are constantly searching for a drug that will effectively manage the progression of DM-induced cardiomyopathy, as none of the existing treatments are sufficient. Of particular interest are antioxidants, coenzyme Q10, microRNAs, substances that counteract increased cellular inflammation and modulators of various signalling pathways.

First of all, because oxidative stress is one of the major pathophysiological pathways contributing to the development of DCM, promoting antioxidant defences appears to be a reasonable treatment goal. Reducing the amount of ROS helps attenuate the oxidative stress and thus ameliorates fibrosis. Coenzyme Q10 is an essential cofactor for ATP production in the respiratory chain. In trials on rats, it lowers blood glucose levels and directly and indirectly affects the oxidative stress as it neutralises and scavenges ROS and decreases NF-κB stimulation resulting in lowering proinflammatory factors, such as TNF-α [125,126]. It also ameliorates collagen deposition in the myocardium, thus reducing cardiac fibrosis. Similarly, vitamin E is a crucial antioxidant that protects against ROS-induced lipid peroxidation. In contrast to coenzyme Q10, it does not lower blood glucose or cholesterol levels; however, animal studies showed it has an anti-atherogenic effect in coronary vessels [127]. That outcome was caused by the reduction in oxidative stress as vitamin E decreases myocardial 8-iso PGF2α levels, which is a specific and sensitive quantitative index of oxidative stress in vivo [128]. As these findings suggest the efficacy of vitamin E supplementation in patients with DM1, it may have similar effects in DM2 as well [127,128]. Furthermore, metallothionein is also such a protein. It scavenges free radicals, therefore reducing oxidative stress, and regulates zinc intracellular concentration, which is a protective factor in the heart as its deficiency leads to coronary artery occlusion and increased cardiac lipid peroxide levels [129]. Hence, both expression of metallothionein and zinc supplementation seem crucial to provide protection from the development of DCM. Moreover, oxidative stress can be addressed by decreasing the NF-κB activation and reducing proinflammatory factors. This is how tempol attenuates cardiomyocyte fibrosis and apoptosis and therefore lowers the risk of DCM development [130,131]. There are also natural antioxidants, such as resveratrol, which are present in many food products, f.ex, red wine. It has a protective effect on the heart due to SIRT1 activation and improving function of the mitochondrion. Resveratrol reduces accumulation of asymmetric dimethylarginine, which is an endogenous inhibitor of nitric oxide synthases, and whose accumulation results in cardiac and mitochondrial dysfunctions. [132] Similarly, the flavonoids, a group of polyphenols present in many fruits and vegetables, have antioxidant and chelating properties. They have been shown to activate the Nrf2 pathway and inhibit the NF-κB pathway, consequently reducing inflammation and oxidative stress [133].

As mentioned earlier, SERCA2a plays a crucial role in Ca^2+^ handling in the heart. Istaroxime has a dual mechanism of action, which consists of the ability to inhibit Na^+^/K^+^ ATPase and enhance the activity of SERCA2a ATPase. The second is achieved by attenuating the inhibitory effect of phospholamban on SERCA2a, without inducing spontaneous Ca^2+^ release from the sarcoplasmic reticulum. The development of a SERCA2a-activating molecule represents a promising strategy for the treatment of HF and DCM [134].

Some drugs that are already used in treating other conditions may be a beneficial therapeutic strategy in DCM. α-lipoic acid, prescribed for treating diabetic neuropathy, upregulates the H_2_S-synthesising enzymes, therefore increasing H_2_S production and resulting in cardioprotection [135]. There are also medicaments for treating other heart conditions that seem advantageous in DCM. According to the study conducted on mice, ivabradine, a blocker of the hyperpolarisation-activated sodium channel used in patients with HF and chronic stable angina, inhibits the expression and activity of MMP that improves cardiac function. MMP is a proteolytic enzyme remodelling the extracellular matrix and playing a role in the inflammatory response. Ivabradine also results in the deactivation of caspase 3 and the activation of NF-kB that contributes to inhibiting the cardiomyocyte apoptosis [136]. Furthermore, trimetazidine (TMZ) improves myocardial substrate utilisation by shifting energy production from FFA to the more energy-efficient glucose oxidation pathway. TMZ prevents myocardial ischemia but has no effect on myocardial oxygen consumption or blood flow and does not negatively affect cardiac inotropism. It maintains appropriate levels of phosphocreatine (PCr) and ATP in cells and improves the low PCr/ATP ratio. In this way, it contributes to the regeneration of mitochondrial oxidative phosphorylation and phosphocreatine resynthesis. TMZ has anti-apoptotic properties and reduces the concentration of free radicals that cause heart damage during ischaemic events. TMZ also improves the function of the vascular endothelium. Thus, TMZ increases the contractile function of the heart muscle. Studies have shown that trimetazidine added to standard HF treatment improves the NYHA functional class, increases exercise efficiency, improves patients’ quality of life and has a beneficial effect on the echocardiographic parameters of LV function in patients with HF secondary to DCM [137]. In turn, ranolazine exerts a protective function in DCM via the NOTCH1/NRG1 signal pathway. The Notch homolog 1 (NOTCH1) pathway is a signalling cascade that participates in maintaining the pluripotency of stem cells in various tissues and thus plays a vital role in embryonic development and cell renewal in adults. NOTCH1 binds to the neuregulin 1 (NRG1) promoter region and directly regulates its transcription. The NOTCH1/NRG1 pathway promotes cardiomyocyte proliferation and therefore may inhibit apoptosis. NRG1 has been shown to be impaired in a DCM model, suggesting that NRG1 plays an important role in DCM development. Ranolazine-induced NOTCH1 activates NRG1 and inhibits the downstream apoptosis-related pathway [138]. Moreover, Ranolazine acts by inhibiting the late inward sodium current, resulting in a decrease in intracellular Ca^2+^ concentration due to the enhancement of the Na^+^-Ca^2+^ exchanger by the sodium gradient. As a result, ranolazine improves Ca^2+^ handling and alleviates myocardial relaxation disorders and diastolic dysfunction. Ranolazine also inhibits fatty acid oxidation and improves the efficiency of glucose oxidation [139].

An entirely new approach to treating DCM is to evaluate the potential of microRNAs. These molecules are gene expression modulators that play a vital role in transcriptional and post-transcriptional modifications [140]. microRNAs influence oxidative stress, inflammation, fibrosis, apoptosis, cardiac muscle calcium metabolism, the synthesis of neurohormones and the functioning of mitochondria [141]. The mechanisms modified by microRNAs influence the development of DCM. It has been shown that microRNAs affect the expression of histone deacetylase and DNA methyltransferase, as well as histone H2A, which have impact on the processes of transcription and translation [142]. Studies have shown that blood levels of circulating microRNAs vary depending on the stage of DCM. Analyses of the changes in microRNA blood levels and those in the myocardium and identification of miRNA-regulated pathways will help discover new ways to treat DCM [143].

These methods of treatment are not established yet because more research still needs to be conducted to evaluate their effectiveness and safety for the patients. Table 2 below summarises the findings described in this section.

## 5. Personalised Therapy

International guidelines recommend a personalised approach to control glucose levels. Numerous patient-specific factors should be taken into account while determining the patient’s glycaemic targets. These are age; duration of DM; presence of comorbidities, such as kidney disease and CVD; risk of hypoglycaemia; and financial status. Hypoglycaemia is the most common adverse reaction of glycaemic control. The prevention of such events results in improved overall health and a smaller number of hospitalisations. Therefore, young and healthy patients may have more strict glycaemic goals in comparison to the elderly ones or those with many comorbidities, because they will better tolerate that condition. Furthermore, taking into consideration the patient’s lifestyle and treatment preferences results in better compliance. Similarly, active patient involvement plays a vital role in effective DM management [144].

On the one hand, individualised glycaemic control has proven to be the most effective. On the other hand, however, it is not without numerous challenges. First of all, the more comorbidities, the more difficult it is to tailor appropriate therapy. The patient should also be well educated about one’s illness. That requires time and patience from both sides. Moreover, good glycaemic control depends on compliance and regular follow-ups. That in turn pivots on the patient’s psychosocial well-being. Stress, depression and family support influence effective DM management [144].

## 6. Conclusions

DCM is an important cause of HF. We have outlined the complex pathophysiology and treatment modalities of DCM. Among the aetiologies of DCM are abnormalities in biochemical pathways, ranging from impaired glucose/fatty acid metabolism, abnormal signalling involving calcium ions, increased inflammation and oxidative stress, which cause fibrosis, stiffness and myocardial hypertrophy. Early diagnosis of DCM is extremely important to halt the progression of the disease. An assessment of risk factors, a complete medical history and a physical examination are useful in decision-making when choosing diagnostic tests. The symptoms of DCM vary and result from the progression of asymptomatic systolic dysfunction to systolic dysfunction and symptomatic HF. In patients with advanced systolic disease, beta-blockers, ACEI/ARBs and MRAs are the mainstay of treatment, although the risk of electrolyte disturbances must be considered. Antiglycaemic drugs also play a very important role by providing effective glycaemic control. GLP-1 agonists and SGLT2 inhibitors are currently the most effective treatments for HF associated with DM. New therapies are being developed, including microRNA therapies, antioxidants and modulators of signalling pathways, to treat and prevent DCM. However, additional research into new therapies to reduce the risk of DCM development and progression should continue.

## Figures and Tables

**Table 1:** Summary of lifestyle recommendations for adults with diabetes mellitus.

Factor	Recommendation	References
Physical activity	≥150 min of moderate- to high-intensity aerobic physical activity (e.g., swimming or cycling) per week 2–3 sessions of resistance exercise per week Balanced training is recommended for elderly people with diabetes mellitus Prolonged (>30 min) sitting should be discontinued	[45]
Daily calories intake	Adjust caloric intake according to gender, age and physical activity (during adulthood, energy requirements decrease by ≈70–100 calories with each decade) Consume an average of about 30 kcal/kg body weight per day Avoid a body mass index ≥ 30 kg/m^2^ and <18.5 kg/m^2^; patients with a body mass index of 18.5–24.9 kg/m^2^ have the lowest risk of death A caloric deficit of 250–500 kcal/day should be implemented in all people with overweight/obesity to promote weight loss (0.5–1.0 kg/week)	[45,50,51]
Carbohydrates	The proportion of carbohydrates in the diet should provide about 45–60% of total kilocalories per day Prefer foods with a low glycaemic index < 55	[50,51]
Proteins	The proportion of protein in the diet should provide about 15–20% of total kilocalories per day (usually 1–1.5 g/kg body weight per day, limit protein intake to <0.8 g/kg body weight in patients with chronic kidney disease)	[50,51,52]
Fats	The proportion of fats in the diet should provide about 20–35% of total kilocalories per day The intake of saturated fatty acids should be reduced to 7–9% of total kilocalories per day The intake of trans fats should be reduced Consumption of foods rich in long-chain omega-3 polyunsaturated fatty acids, monounsaturated fatty acids and plant stanols/sterols should be preferred	[50,51,52]
Fibre	Patients with diabetes mellitus should consume 30–50 g of fibre from raw vegetables and unprocessed grains daily (including ≥ 30% soluble fibre)	[52,53]
Microelements and vitamins	Possible deficiencies in micronutrients and vitamins should be detected and supplemented	[51,52]
Sodium intake	Individuals with diabetes mellitus should limit salt intake to <5 g per day (<2 g Na)	[54]

**Table 2:** Potential future therapies of cardiomyopathy in patients with DM.

Subject	Mechanism of Action	References
Coenzyme 10	Reduction in blood glucose level Reduction in cholesterol level Attenuates oxidative stress Suppression of inflammation Inhibition of myocardial fibrosis	[125,126]
Vitamin E	Reduction in ROS production Prevention of atherosclerosis in coronary vessels	[127,128]
Metallothionein	Protection against oxidative DNA, protein and lipid damage by ROS Increasing intracellular zinc levels in cardiomyocytes	[129]
Tempol	Attenuating the effects of ROS and vascular complications Inhibiting apoptosis and fibrosis of cardiomyocytes	[130,131]
Resveratrol	Reduction in blood glucose level Improving mitochondrial functions Increasing NO level	[132]
Flavonoids	Activation of antioxidant enzymes Inhibition of oxidases Suppression of inflammation	[133]
Istaroxime	Improvement in myocardial relaxation and contraction Improvement in the diastolic index Increasing systolic blood pressure and cardiac index	[134]
Trimetazidine	Reduction in cardiomyocytes apoptosis Improving energy utilisation Improving the function of vascular endothelium Lowering the concentration of ROS Increasing the contractile function of the heart muscle	[137]
Ivabradine	Inhibition of cardiomyocytes apoptosis Relieving inflammation	[136]
α-lipoic acid and H_2_S	Protecting cardiac function during ischaemia–reperfusion Decreasing oxidative stress	[135]
Ranolazine	Preventing apoptosis Supporting renewal of cardiomyocytes Alleviating myocardial relaxation disorders and diastolic dysfunction. Improving the efficiency of glucose oxidation	[138,139]
microRNA	Acting as a reactive oxygen species scavenger Improving sensitivity Suppression of inflammation, fibrosis and apoptosis Stimulation the synthesis of neurohormones Improving the calcium metabolism of the heart muscle	[140,141,142]

## Data Availability

Not applicable.
